# Editorial: Effect of overweight/obesity on early puberty and related chronic disease risk factors

**DOI:** 10.3389/fendo.2024.1357819

**Published:** 2024-01-25

**Authors:** Reihane Taheri, Spencer D. Proctor

**Affiliations:** Metabolic and Cardiovascular Diseases Laboratory, Division of Human Nutrition, University of Alberta, Edmonton, AB, Canada

**Keywords:** obesity, youth, chronic disease, diabetes, dyslipidemia

## Clinical problem

Obesity is often defined as those with excessive or abnormal body fat that in turn contributes to associated comorbidities; some of these include; Alzheimer’s disease, cardiovascular disease (CVD), respiratory disease, autoimmunity, diabetes, some cancers (prostate, ovarian, breast and colon), non-alcoholic fatty liver disease (NAFLD), and osteoarthritis (1–3). As a result, obesity is ranked as 5^th^ leading cause of death globally (1).

The World Health Organization (WHO) report in 2016 indicated that the prevalence of overweight and obesity in children and adolescents (aged 5-19) quadrupled from 4% in 1975 to 18% in 2016 (2). The exact cause of obesity is multifactorial, but it is the combined result of energy imbalance due to complex interactions between genetic and behavioural risk factors such as diet, physical activity, psychosocial factors (4). Childhood obesity typically associates with emerging medical problems such as the metabolic syndrome (MetS), puberty disorders, NAFLD, as well as sleep apnea during developmental stages (5). Importantly, we know that children with obesity are at higher risk of obesity in adulthood as well as future cardiometabolic diseases (6, 7). There are further implications to youth with obesity including the development of early social and emotional consequences (5, 8). Due to the importance of childhood obesity, *Journal of Frontiers in Endocrinology* has chosen to call this Research Topic to discuss recent findings in the area.

## Energy expenditure and physical activity

Reduced physical activity, especially in children, has been considered as one of the initiating factors contributing to increased prevalence of obesity (9). However, in this Research Topic Le et al. rightly voiced that the relation of physical activity with maintaining healthy weight during adolescence is complicated. For example, it remains to be resolved whether leisure-time physical activity (LTPA) is directly associated with future BMI or fat mass in adolescents. At the same time, vigorous physical activity during adolescence has been shown to be associated with a reduced BMI at early adulthood, but only in boys. These observations highlight that during adolescence, the level of fatness (but not necessarily physical activity) maybe a predictor of future adiposity. It is also critical to appreciate that the relation of physical activity and fat mass varies among boys and girls depending on the pubertal status.

## Non-alcoholic fatty liver disease

Obesity is commonly associated with an increased risk of NAFLD and MetS during adolescence (10, 11). MetS is characterized by high fasting plasma glucose, insulin resistance, dyslipidemia, high blood pressure and obesity. These clinical features are also seen in patients with NAFLD (now also known as Metabolic dysfunction-Associated SteatoHepatitis or MASH). NAFLD is the most common cause of chronic liver disease in children and adolescents that tracks with liver disease in adulthood (3, 10, 12). In this Research Topic, Zhou et al. highlighted that high BMI is perhaps the greatest risk factor for NAFLD incidence in children. Their study found that in children with obesity (prior late puberty), the most significant risk factors for NAFLD included BMI, alanine transaminase (ALT), and age. Interestingly, they found that the greatest risk exposures for NAFLD in children were BMI and ALT. Of course, the early diagnosis of dysglycemia, dyslipidemia and obesity are now critical to protect children from developing both MetS and NAFLD. In terms of dyslipidemia, traditional lipid biomarkers used for MetS diagnosis are high triglycerides (TG), total cholesterol (TC), low-density lipoprotein cholesterol (LDL-C) and low high-density lipoprotein cholesterol (HDL-C). However, plasma TC and LDL-C levels often drop physiologically during puberty (13). For example, in a study in Canada, reported that adolescents with obesity can have relatively normal level of classic lipid levels while still presenting with cardiometabolic risk and high apo-lipoprotein B levels (14). A better understanding of these non-traditional lipid biomarkers during adolescence would be useful in this clinical context. In this Research Topic, Chen et al. compared the association of adolescents with MetS with both traditional and non-traditional biomarkers including apo-B lipoprotein, non-HDL-C, and lipid accumulation product (LAP). Their study highlighted that most of these lipid biomarkers are closely associated with MetS in youth. Curiously, LAP had the closest association with the risk of MetS. LAP can be readily calculated by measuring both the waistline and blood concentrations of TG. Advancing the clinical usefulness of non-tradition lipid biomarkers may indeed be the path by which we can improve our early diagnosis metabolic dysfunction in those youth with obesity.

## Early obesity and hormonal interactions

Childhood obesity is known to affect the onset of puberty during adolescence. Studies have reported that obese girls have a greater susceptibility to precocious puberty in comparison to normal-weight girls, but this association is less clear in boys (8). In this Research Topic, the longitudinal study by Choe et al. found that rapid weight gain during infancy (birth-12 months) and toddlerhood (1-to 3 years old) is not associated with early central precocious puberty (CPP) in boys. In contrast, girls who experienced rapid weight gain during both infancy and toddlerhood had the highest increased risk of (CPP). These findings suggest that early detection of excessive weight gain, especially during infancy, maybe essential to prevent future CPP in girls. However, obesity is not the only risk factor associated with early onset of puberty (15). Here, Tang et al. showed that after adjustment of BMI, dietary patterns, soft drink, feeding pattern and mother’s BMI, short sleep duration, were all associated with early pubertal development in both girls and boys. Researchers attempted to find ways to prevent obesity and precocious puberty in different ways including use of nutrients. One of the beneficial nutrients proposed to be effective in prevention of obesity and obesity-related precocious puberty is (-)-Epigallocatechin-3-gallate (EGCG), the most abundant catechin in tea. In order to find out the mechanism which EGCG affects puberty, Gu et al. assessed the metabolites involved in endocrine-related pathways (estrogen signaling pathway, insulin resistance, and insulin secretion), and signal transduction (PI3K-Akt, MAPK, and Jak-STAT signaling pathways). They found that the potential protective effect of EGCG is related to both endocrine and signal transduction pathways. For example, EGCG can target multiple signaling pathways, including estrogen, PI3K-Akt, MAPK, and Jak-STAT pathways. This study provided foundation for future research using EGCG for treatment of obesity and precocious puberty.

## Final comments

Childhood obesity is a major risk factor for developing chronic diseases in adulthood. The development of obesity in childhood and adolescence is complex and multifactorial. Advancing this field remains critical for us to better understand prevention measures and clinical parameters that will aid in the early detection and resolution of this trajectory.

## Author contributions

SP: Writing – original draft, Writing – review & editing. RT: Writing – original draft, Writing – review & editing.
